# Extraction of phylogenetic network modules from the metabolic network

**DOI:** 10.1186/1471-2105-7-130

**Published:** 2006-03-13

**Authors:** Takuji Yamada, Minoru Kanehisa, Susumu Goto

**Affiliations:** 1 Bioinformatics Center, Institute for Chemical Research, Kyoto University, Gokasho, Uji, Kyoto, 611-0011, Japan

## Abstract

**Background:**

In bio-systems, genes, proteins and compounds are related to each other, thus forming complex networks. Although each organism has its individual network, some organisms contain common sub-networks based on function. Given a certain sub-network, the distribution of organisms common to it represents the diversity of its function.

**Results:**

We extracted such "common" sub-networks, defined as "phylogenetic network modules," using phylogenetic profiles and cluster analysis. The enzymes in the same "phylogenetic network module" have similar phylogenetic profiles and related functions. These modules are shown to be phylogenetic building blocks. Furthermore, the network of the modules illustrated hierarchical feature as well as the network of enzymes involved in the metabolism.

**Conclusion:**

We conclude that phylogenetic network modules are evolutionary conserved functional units in the metabolic network. We claim that our concept of phylogenetic modules provides a more accurate understanding of the evolution of biological networks.

## Background

Bio-systems are constructed by various relationships between genes, compounds and proteins. These elements are intertwined, thus forming a complex network. Such genetic or physical associations contribute to the complicated cellular functions.

In order to characterize whole networks or to find unknown interactions between elements (gene/compound/protein), many research groups have applied the integration of qualitatively different interactions. For example, Rison et al attempted to find unknown functions of genes using genome location, sequence similarity, and relative position in the metabolic network [1], and Yamanishi *et al *similarly attempted to apply a kernel method [2]. Such integrative methods infer common features and new categories of genes. Classically, such new categories were determined manually using expert knowledge. The KEGG metabolic pathway [3] is one such example. On the other hand, recent studies have attempted to define new categories automatically using comprehensive data sets such as microarray data and metabolic networks [4]. Such analyses can classify elements by globally assessing many relationships. Many research groups advocate various functional categories of genes and proteins, such as transcriptional clusters [5,6], protein complexes [7], metabolic network [3,8], and others [9,10].

From the perspective of graph theory, it is suggested that networks composed of genes and proteins should have modularity. Ravasz *et al *suggested a hierarchical network model incorporating modularity in the metabolic network [11]. Following this work, studies were attempted using the modularity in various networks and topological features [12]. However, these topological investigations were not based on phylogeny; Snel *et al *pointed out problems in which they found conflicts between evolution and function [13].

In order to resolve these conflicts, the main purpose of our research is the extraction of the phylogenetic primary units of the metabolic network for understanding the evolutionary process. To our knowledge, we were the first to attempt to extract evolution-based enzyme modules. In our previous work, we attempted to extract phylogenetic units using prokaryotic genomes [14]. Here, we expanded the analysis by adding eukaryotic data, and by considering the topological properties of the network of phylogenetic units.

The metabolic network was considered not only to be composed of several particular categories, but also to be a large network consisting of chemical compounds connected by enzymes. For example, KEGG/PATHWAY is a collection of manually drawn pathway maps. However, we can regard all of these pathway maps as a "metabolic network." Our analysis is based on the KEGG/PATHWAY database, which integrates the metabolic network from many organisms. Therefore, in this paper, we use the term "metabolic network" to refer to this integrated metabolic network. Therefore, some parts of the metabolic network are found only in particular organisms, and the number of organisms varies depending on the sub-networks. The phylogenetic profile is very useful to deal with such diversity of enzymes. The phylogenetic profile of an enzyme is the string that encodes the presence or absence of the enzyme in the fully sequenced genome [15].

**Figure 1:**
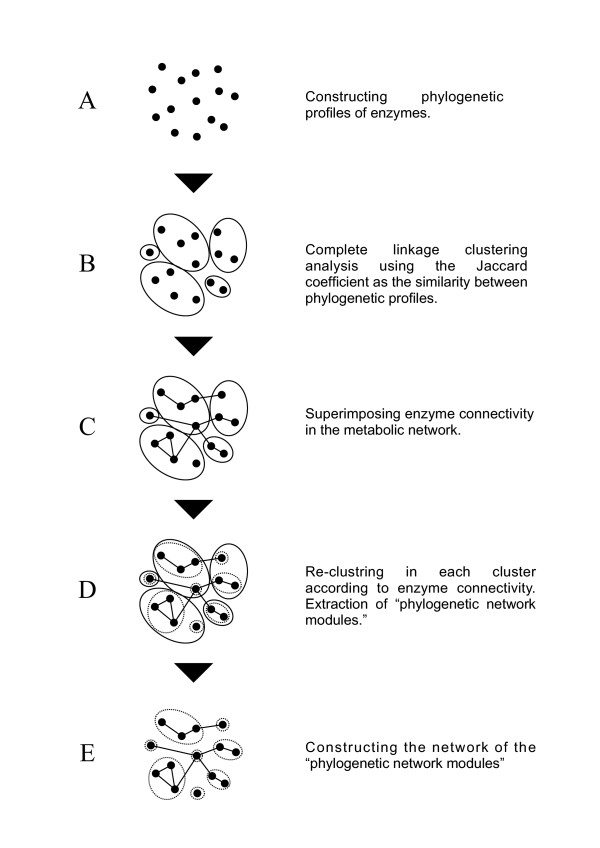
**Overview of extracting the phylogenetic network modules**. Black dots represent enzymes, and lines between the dots represent enzyme connectivity in the metabolic network (common metabolites in the enzyme reactions). Circles with solid line are the enzymes cluster from the result of complete linkage clustering. Circles with doted line are the phylogenetic network module.

Using the enzyme connectivity in the metabolic network and the similarity between the phylogenetic profiles of the enzymes, we extracted enzyme modules and define them as "phylogenetic network modules." The enzymes in the same phylogenetic network module have similar phylogenetic profiles and are located close to one another in the metabolic network. In other words, these enzymes behave in a similar way in the evolutionary process of the metabolic network. Furthermore, we found a hierarchy of phylogenetic network modules. The method overview is summarized in Figure 1. Our result fits the concept of the network model of Ravasz *et al *[11]. Our phylogenetic network modules are based on the simultaneous behaviour of multiple enzymes in the evolutionary process of the metabolic network. This allows for the detailed understanding of metabolic network evolution.

## Results

### Pathway distance and the similarity of phylogenetic profiles

**Figure 2:**
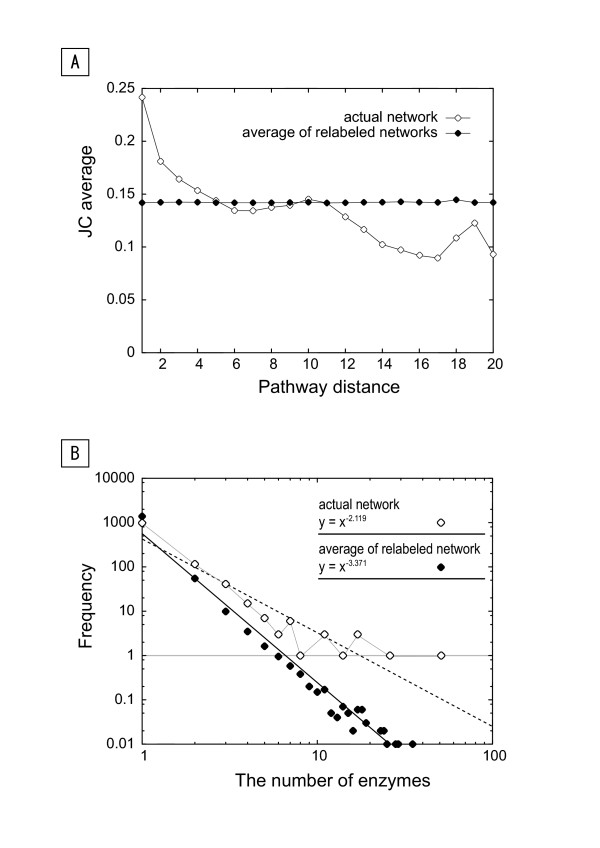
**A: Relationship between the average of the Jaccard coefficient (JC) and the pathway distance, B: The distribution of the number of enzymes in a "phylogenetic network module"**. A: This figure indicates the transition of the JC average according to pathway distance. Pathway distance is defined as the smallest number of steps between two enzymes in the metabolic network. The white and black circles correspond to an actual network and the average of relabeled networks, respectively. The obvious difference appears in the area where pathway distance is less than 5 steps. B: This figure shows the difference between the actual and the average of relabeled networks in terms of the number of enzymes in a module. White dots correspond to the actual modules, and the black ones correspond to the modules generated from relabeled networks. The equations in the upper portion give the slope of the line fitted by the least-squares method.

Figure 2-A illustrates the negative correlation between pathway distance and the similarity of phylogenetic profiles (white points). We use the Jaccard coefficient (JC) as the similarity score of phylogenetic profiles of enzymes, and pathway distance is defined as the smallest number of steps between two enzymes in the metabolic network. The figure shows that the longer the pathway distance, the smaller the average similarity. In fact, two adjacent enzymes in the network have the highest average similarity score. We generated randomly relabeled networks 100 times, and plotted their average for comparison (see Method section). Contrary to the actual metabolic network, no pattern was observed in the JC average of relabeled networks (black points in Fig. 2-A). The JC average of relabeled networks is constant approximately 0.14, which is equal to the average similarity score when comparing all profiles against each other. We also performed the same analysis using correlation coefficient (CC) as a different similarity measure, which resulted in a similar trend to that of JC (see additional file 1).

### Distribution of the number of enzymes included in a module

The white points in Figure 2-B indicate the frequency of the "phylogenetic network modules" according to their sizes (the number of enzymes). The horizontal axis represents the number of enzymes in a module, and the vertical axis represents its frequency. In total, 1179 modules were extracted, but over 900 modules contain only one enzyme. There are a few large modules, and many small modules. Similar results were obtained using different distance measures such as correlation coefficient and Hamming distance (data not shown).

Furthermore, in order to prove that the enzymes form module structures in the metabolic network, we compared the frequency with the average of 100 randomly relabeled networks, shown as the black points in Figure 2-B. The difference of slope indicates that larger modules tend to appear more frequently in actual networks; that is, they hardly appear in the average of relabeled networks. This implies that enzymes with similar phylogenetic profiles tend to aggregate in the network, and that phylogenetic module structures are indeed present in the metabolic network.

### Comparing modules with the categories in the KEGG/PATHWAY database

KEGG/PATHWAY is a collection of manually drawn diagrams called the KEGG reference pathway diagrams (maps), each corresponding to a known network of functional significance. These diagrams are drawn manually representing particular functions of biological processes. We mapped our phylogenetic network modules onto these diagrams.

**Figure 3:**
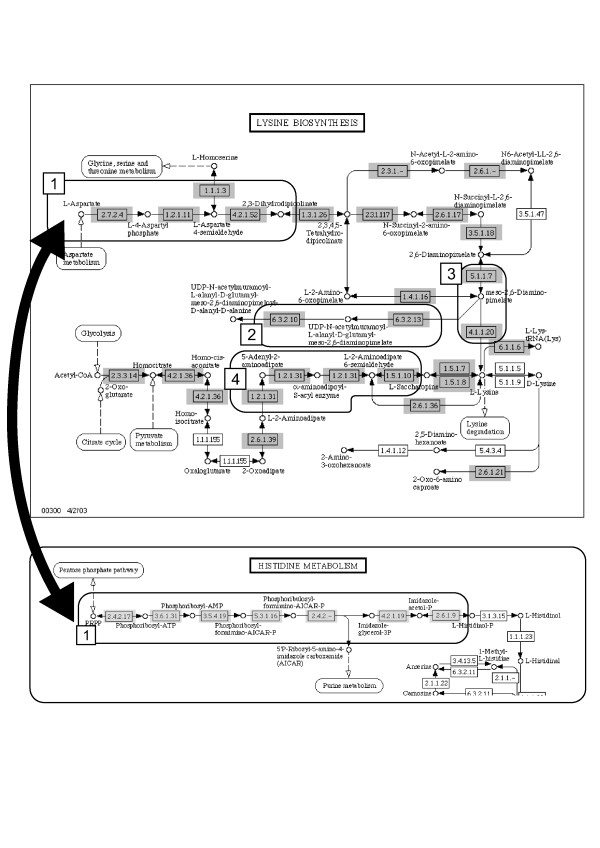
**Example of the network module**. This figure shows the lysine biosynthesis pathway. Enzymes surrounded by the solid lines represent parts of particular modules. The other gray colored enzymes correspond to singleton modules, which are those that contain only one enzyme. Lysine biosynthesis includes multiple modules, which also spread to the other map categories.

The upper portion of Figure 3 illustrates an example of mapping phylogenetic network modules onto a diagram (Lysine biosynthesis). Gray colored enzymes are contained in the organisms in the KEGG database. Enzymes surrounded by a solid line represent a part of a particular phylogenetic network module, each corresponding to different phylogenetic and functional features.

Module 1 is contained in many organisms, spreading to several amino acid biosynthesis pathways such as histidine metabolism (lower part of Figure 3). Module 2 is specialized to prokaryotes, so it links to the prokaryote-specific Peptidoglycan biosynthesis pathway. Module 3 is also contained in relatively many organisms, and module 4 is specialized for eukaryotes. Almost all of the diagrams in KEGG/PATHWAY include multiple phylogenetic network modules, and some modules spread to many diagrams. In fact, the lysine biosynthesis pathway contains several modules, and module 1 and 2 spread to other diagrams. Thus, using our phylogenetic network modules, we could detect phylogenetic relationships between known functional categories (across pathways).

### Global network of phylogenetic network modules

**Figure 4:**
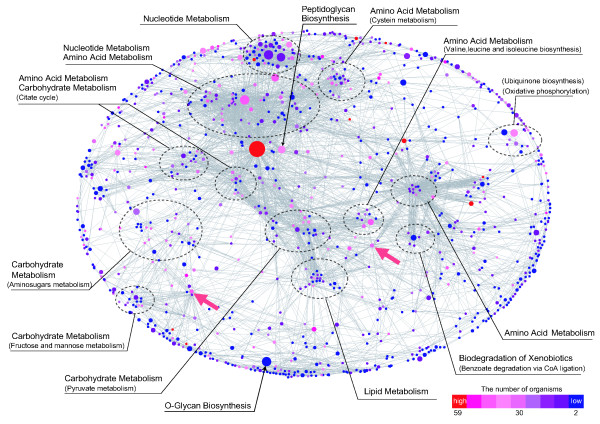
**The largest component of the module network**. Each node and edge represents an individual module and reaction on the network. The size and the color of the node correspond to the number of enzymes and the number of organisms included in the module, respectively. The dotted circles indicate the locations where modules relatively aggregate. Modules pointed out by the red arrows are obvious linkers, which seem to play a role in connecting modules.

Figure 4 illustrates the largest component of our global module network which includes 1130 of the 1179 modules. This is the reconstruction of a whole metabolic network by our phylogenetic network modules, so each node corresponds to a module, and each edge corresponds to the relationship between them. The size of a node indicates the number of enzymes included in the module, and its color indicates the number of organisms associated with the module.

Three features are conspicuous in this figure. The first is a large central module. All of the modules seem to gather around the largest red module as a network core. The big module contains 51 enzymes that spread to a large part of nucleotide metabolism, and a part of amino acid metabolism. Furthermore, this is contained in almost all organisms so that it is not surprising that the module constructs a core of the metabolic network. This module connects other modules with each other and sustains the whole network. Additionally, relatively large modules are also observed in parts of the metabolic network: some glycan related pathway diagrams for eukaryotes and a part of peptidoglycan biosynthesis for bacteria.

As a second feature, modules also aggregate. In Figure 4, some of the modules tend to assemble together into clusters. The dotted circles in the figure indicate the locations of what we call "super-modules," which are modules that are relatively aggregated. The relationship between modules in this figure is based on the connectivity of existing enzymes in the metabolic network, so the "super-modules" represent modules of related functions even though some spread to multiple pathway diagrams (only representative diagrams are annotated in Figure 4). For example, the super module structure just above the network core (the biggest red module) in Figure 4 is composed of many diagrams of Amino acid metabolism, and it contains many links to other modules. Basically, the enzymes in this super-module are highly conserved and are closely related to those in the network core. Thus, we claim that enzymes in these modules use many metabolites synthesized in the core structure and that they also supply many metabolites to the other modules. The third feature is that "linker" modules are scattered around the network. As a topological feature, they have relatively low clustering coefficients compared to the other modules with the same degree (see Methods section). Therefore they do not belong to any particular module cluster, and instead, link module clusters to each other. We found 30 linker modules by the criterion defined in the Methods section. Biologically, linker modules tend to be intermediates for the input and output compounds of functional modules. For example, the linker module indicated by the right arrow in Figure 4 connects Amino Acid Metabolism, Lipid Metabolism, as well as a few others. This module is composed of three enzymes (3-hydoroxyacyl-CoA dehydrogenase, acyl-CoA dehydorogenase and enoyl-CoA hydrase) that catalyze reactions between acetoacetyl-CoA and crotonyl-CoA. This reaction chain produces acetyl-CoA in Amino Acid Metabolism and consumes it in Lipid Metabolism, thus linking these two pathways together. As another example, the module indicated by the left arrow in Figure 4 contains three enzymes, glucose-6-phosphate isomerase, phosphoglucomutase and glucose-6-phosphate 1-dehydrogenase. Although these enzymes play roles in Glycolysis, they connect compounds to other Carbohydrate Metabolism pathways such as Aminosugars metabolism and Fructose and mannose metabolism.

## Discussion

### Functionally related phylogenetic module

Known functional modules do not completely coincide with evolutionary modules. Snel *et al*. investigated whether known functional modules are also evolutionary modules and suggested that all the members of the same functional module do not have co-evolutionary tendencies [13]. This means that evolutionary modules are not in complete agreement with functional modules.

Basically, we agree with Snel's opinion because of the difference of enzyme distributions in the phylogenetic network modules due to the addition of enzyme connectivity in the metabolic network. Before adding information of enzyme connectivity, enzyme clusters were constructed using only the similarity between phylogenetic profiles (Fig. 1B). After that, enzymes were re-clustered within each cluster using connectivity in the metabolic network (Fig. 1C,D,E). We call these sub-clusters phylogenetic network modules. Obviously, the number of enzymes in a phylogenetic network module was less than those in the original clusters. It is clear that enzyme connectivity in the metabolic network subdivides the enzyme clusters. This explains precisely the conflict between functional modules and phylogenetic modules. By definition, the phylogenetic network modules are the evolutionarily conserved and functionally related enzyme modules in the metabolic pathway. As a result, we claim that the phylogenetic network module is the basic functional unit in the metabolic pathway.

### Modularity and hierarchy in the evolutionary process of the metabolic network

Historically, there have been many analyses of network evolution [16], which has led to advanced theories on network evolution. Two major assumptions are generally thought to be the main contenders. One is a retrograde model [17], and the other is a patchwork model [18]. In the retrograde model, network evolves "backwards" from a key metabolite. This model expands the network due to the acquirement of new enzymes, which synthesize a molecule used up in the environment from other molecules. On the other hand, in the patchwork model, network evolution is based on the concept that enzymes exhibit broad substrate specificity and catalyze multiple reactions. Such enzymes with broad specificity form reaction chains to a key metabolite. The important thing is that those two models are not mutually exclusive, and they are reviewed in [19].

In any case, these evolutionary models are based on the relationship between enzymes and substrates (metabolites). In this paper, phylogenetic network module corresponds to one function which is a group of chemical reactions catalyzing metabolites into others. Correspondingly, we consider the enzyme module as an extended enzyme function in these models. We claim that the concept of representing multiple nodes as a single node, the enzyme module in this case, is important for understanding the evolutionary process of metabolic network. A similar concept for the network integrating other types of relations was proposed in [20]. Our methodology is based on this concept, and our results support the utility of it.

**Figure 5:**
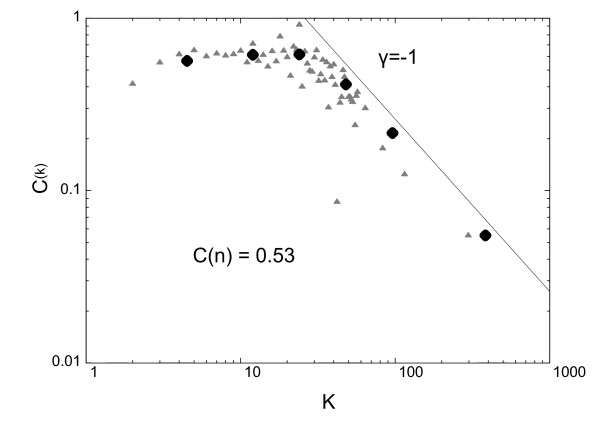
**Topological feature of the module network**. This plot illustrates the dependence of the clustering coefficient on the node's degree. The horizontal axis is node's degree, and vertical axis is the average of clustering coefficient of the nodes which have same degree. Clustering coefficient of node *i *is defined as *C*_*i *_= 2*n*_*i*_/*k*_*i*_(*k*_*i*_-1), where *n*_*i *_denotes the number of links connecting the *k*_*i *_neighbors of node *i *to each other. Black dots represent the binning node according to the C(k) average in each power of 2.

It is well known that the metabolic network is a hierarchical network. When a particular network has a hierarchical feature, its plot (where the vertical axis is the clustering coefficient (C(k)) and the horizontal axis is the node's degree) gives a power-law distribution (γ = -1) [11]. In this paper, we constructed a network of phylogenetic network modules, which we plotted. Interestingly, it had just such modular and hierarchical features as illustrated in Figure 5. This indicates that our notion of evolutionarily and functionally conserved modules explains the hierarchical features suggested by Ravasz *et al *[11], who illustrated the hierarchical structure of the metabolic network using topological properties. Our result suggests that the relationship between enzymes based on the similarity of phylogenetic profiles is one of the factors forming the hierarchical structure.

### Future direction and perspective on network modularity

We hierarchically clustered enzymes to extract phylogenetic network modules. According to this method, enzymes are allocated to a particular module. However, there are some cases where enzymes could belong to multiple functional modules. For example, there are many enzymes catalyzing reactions related with pyruvate or acetyle-CoA. These enzymes have numerous relationships to other enzymes in the metabolic network. In such cases, the phylogenetic relationships between them are complicated, and enzyme allocation to a particular module is difficult. While these enzymes play an important role, it is difficult to divide into a particular module. Given this problem, it may be important to somehow allow enzyme redundancy in the modules, or remove these enzymes from the network. In a module network, linker modules correspond to this case. These modules have a characteristic feature of connecting many modules (module groups), so it will be difficult to determine particular module groups for linker modules.

Although our work focused on enzymes, the components of the metabolic network consist of both enzymes and chemical compounds. There have been a few attempts to investigate the relationships between chemical compounds and phylogeny. Hattori *et al *defined the similarity between compounds in the metabolic network, and generated clusters according to this similarity measure [21]. They also attempted to compare these clusters with operon like structures. However, since the operon data they used was very limited, it was insufficient to attempt phylogenetic analysis. Our phylogenetic network modules are suitable for the analysis, and comprehensive analysis of chemical structures and their evolution is our next research focus.

## Conclusion

We extracted "phylogenetic network modules" from the metabolic network. We claim that these modules are the evolutionary building blocks as well as the basic functional units of the metabolic network. Furthermore, we showed that the module network has the hierarchical character, which is also conserved in the enzyme network of metabolism.

Barabasi *et al *illustrated the hierarchical structure of the metabolic network using topological property. Our result suggests that the relationship between enzymes based on the similarity of phylogenetic profiles is one of the factors forming the hierarchical structure. Other biological systems, such as protein-protein interaction networks, have been reported to have the similar topological property. Thus, the phylogenetic relationship may be a foundation of network evolution including other biological systems.

## Methods

### Construction of the phylogenetic profiles and the network of enzymes

A phylogenetic profile is a bit string that encodes the absence (0) or presence (1) of an enzyme in fully sequenced genomes. We utilized KEGG Orthology (KO) for constructing the phylogenetic profiles. KO is a database of ortholog groups that are defined manually according to the similarity of amino acid sequences, as well as bidirectional best hit information in pairwise genome comparison, and annotated functions of genes in KEGG pathways [3]. Therefore, some proteins with low sequence similarity may be put into the same ortholog group (the same node in the KEGG pathway). In this study, we used KO entries for enzyme genes annotated in the KEGG/PATHWAY database. Phylogenetic profiles were calculated using the KO entries constructed from 174 fully sequenced genomes. To reduce the effect of bias in the organism distribution, these 174 genomes were merged into 59 taxa (11 eukaryotes, 36 bacteria and 12 archaea) according to the NCBI taxonomy [22]. That is, logical OR was applied on the phylogenetic profiles of the organisms of the same taxa. As a result, we obtained 1672 phylogenetic profiles consisting of 59 bits.

The KEGG/PATHWAY database stores metabolic and regulatory pathway information with their functional classification as XML files [3]. The pathway information (the network of enzymes) is described as a collection of binary relationships of enzymes. The binary relationship is defined when the two enzymes are adjacently located on the pathway diagram. We extracted the binary relationships of the enzymes from the KEGG/PATHWAY database and treated them as a network or a graph of enzymes. All the data is accessible from the KEGG website [23].

### Similarity measure between phylogenetic profiles

Similarity measures between phylogenetic profiles are required in cluster analysis. We adopted the Jaccard coefficient (JC) [24] and the correlation coefficient (CC) as the similarity measures of phylogenetic profiles. The Jaccard coefficient between profile *A *and *B *is defined as *A *∩ *B/A *∪ *B. A *∩ *B *is the number of organisms which include enzyme *A *and *B*, and *A *∪ *B *is the number of organisms which include either enzyme A or B. The correlation coefficient used was Pearson's product moment correlation coefficient using a phylogenetic profile as a vector.

### Pathway distance and the corresponding average of Jaccard coefficient

Pathway distance is defined as the smallest number of steps between two enzymes in the metabolic network. We calculated the pathway distance of all against all enzymes in KEGG/PATHWAY and further calculated the JC average for each pathway distance. We also generated 100 relabeled enzyme networks as a control. In this paper, we define a relabeled network as one where the phylogenetic profiles are randomly re-assigned to enzymes in the network with the topology unchanged. The JC average of each pathway distance was calculated for each relabeled network. The average was then taken of all the JC averages of each relabeled network.

### Extraction of the "phylogenetic network module"

An overview of the extraction of the "phylogenetic network module" is illustrated in Figure 1. We performed a complete linkage clustering of the enzymes (KO entries) in our dataset (Fig. 1B) based on their phylogenetic profile similarities. To determine the threshold of this clustering, we utilized the distribution of all-against-all similarities between the phylogenetic profiles of enzymes. From the distribution, we estimated the top 1, 2.5 and 5 percentile points of the similarities as the significant point (each JC corresponds to 0.76, 0.64 and 0.51 respectively, see additional files 2-3). After that, by linking the enzymes according to their connection in the metabolic pathway (Fig. 1C), some "phylogenetic network modules" were extracted in each cluster (Fig. 1D). That is, each group of linked enzymes within each cluster was extracted as a single module. There was no difference in the distribution shape of the module sizes for different significant points. All figures in this paper are based on the 5 percentile point. The same operations were applied to the 100 relabeled networks, and their distribution of the average number of enzymes per module was obtained.

### Network of modules

Finally, we constructed a network of modules, where each node represents a module, and each edge represents the chemical compounds between the enzymes contained in the corresponding modules (Fig. 1E). To infer the structure of the module network, we investigated the relationship between each node's degree and its clustering coefficient. The degree of node *i *is the number of connected nodes. The clustering coefficient of node *i *is defined as *C*_*i *_= 2*n*_*i*_/*k*_*i *_(*k*_*i*_*-l*), where *n*_*i *_denotes the number of links connecting the *k*_*i *_neighbors of node *i *to each other.

Based on the clustering coefficient and the degree, linker modules were selected. These linker modules tend to have high degree and relatively low clustering coefficients compared with other modules of the same degree. We defined the condition of the linker as having degree > 35, and the deviation of the clustering coefficient of the same degree being < -0.1.

## Authors' contributions

TY and SG contributed to develop methodology and to assess the biological significance of the results. MK supervised the project. All authors read and approved the final manuscript.

## Supplementary Material

Additional File 1 The comparison of Jaccard coefficient with correlation coefficient as the similarity measure of the phylogenetic profile. Click here for file

Additional File 2 The distribution of the number of enzymes in a phylogenetic network module using Jaccard coefficient with three different thresholds. Click here for file

Additional File 3 The distribution of the number of enzymes in a phylogenetic network module using Correlation coefficient with three different thresholds. Click here for file
