# Supplementary material for: Extraction of phylogenetic network modules from the metabolic network
Source: BMC Bioinformatics. 2006 Mar 13;7:130. doi: 10.1186/1471-2105-7-130 (PMC1501048; doi:10.1186/1471-2105-7-130)

Additional file 1

The comparison of Jaccard coefficient (JC) with Correlation coefficient (CC) as the similarity measure between phylogenetic profiles.

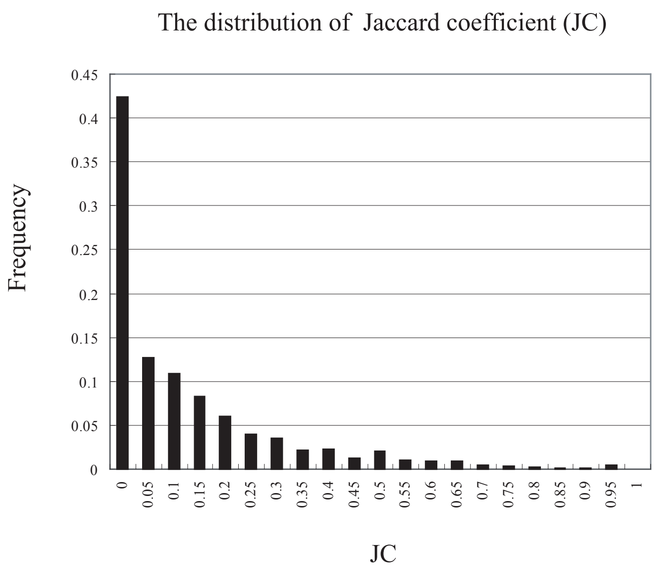

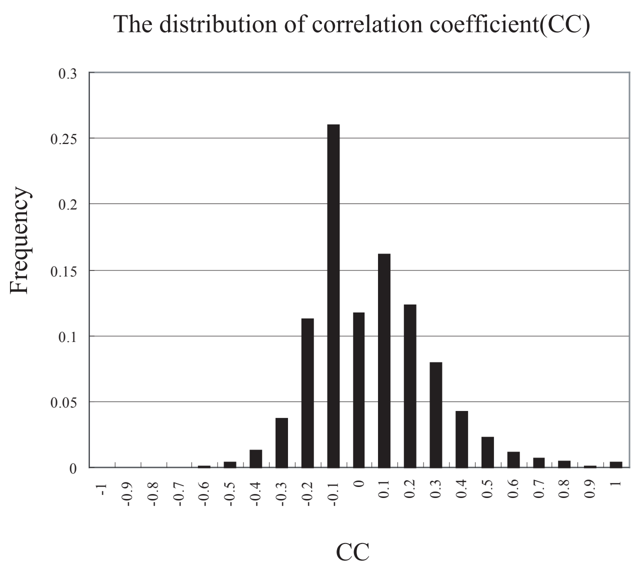

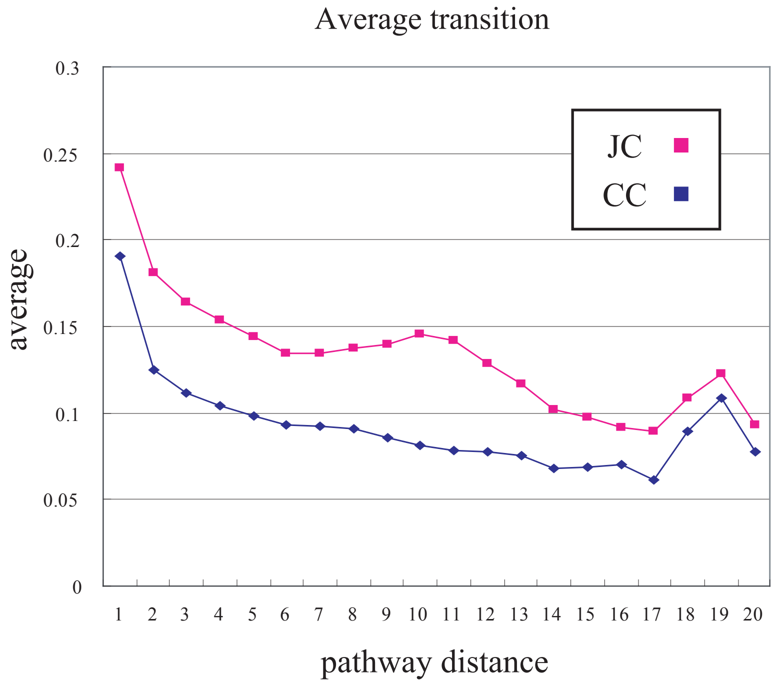

Supplement: Additional File 1 — The comparison of Jaccard coefficient with correlation coefficient as the similarity measure of the phylogenetic profile. [file 1471-2105-7-130-S1.pdf]
