# Supplementary material for: Extraction of phylogenetic network modules from the metabolic network
Source: BMC Bioinformatics. 2006 Mar 13;7:130. doi: 10.1186/1471-2105-7-130 (PMC1501048; doi:10.1186/1471-2105-7-130)
Supplement: Additional File 2 — The distribution of the number of enzymes in a phylogenetic network module using Jaccard coefficient with three different thresholds. [file 1471-2105-7-130-S2.pdf]

Additional file 2

The distribution of the number of enzymes in a "phylogenetic network module" using Jaccard coefficient(JC) with three different thresholds (top 1, 2.5 and 5 percentiles).

P(0010)

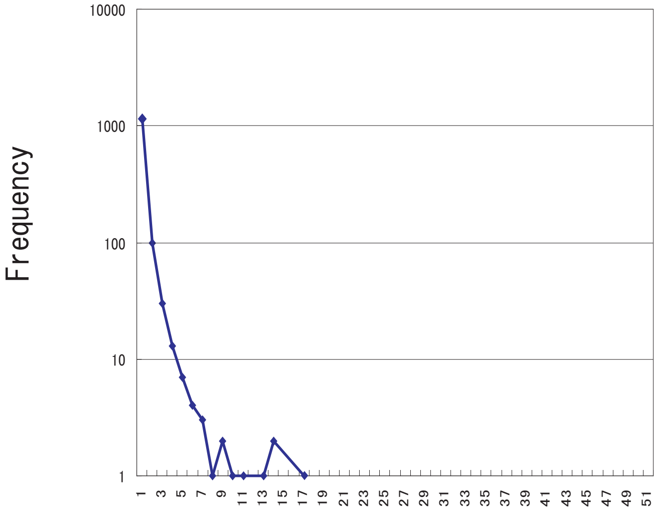

#Class	#Freq
1	1149
2	99
3	30
4	13
5	7
6	4
7	3
8	1
9	2
10	1
11	1
13	1
14	2
17	1

P(0025)

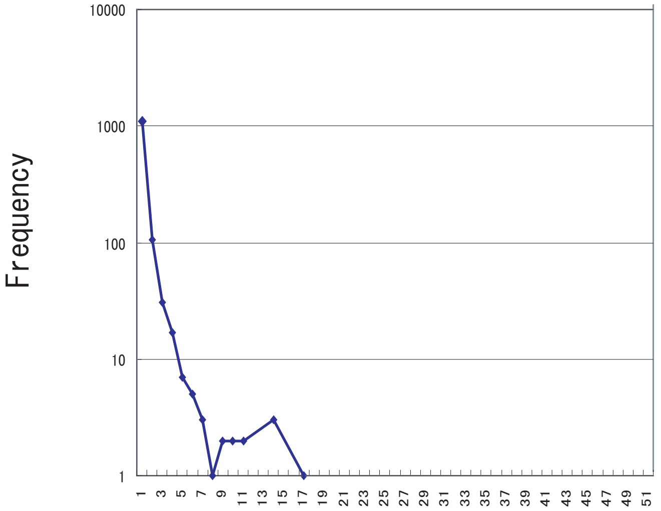

#Class	#Freq
1	1090
2	105
3	31
4	17
5	7
6	5
7	3
8	1
9	2
10	2
11	2
14	3
17	1

P(0050)

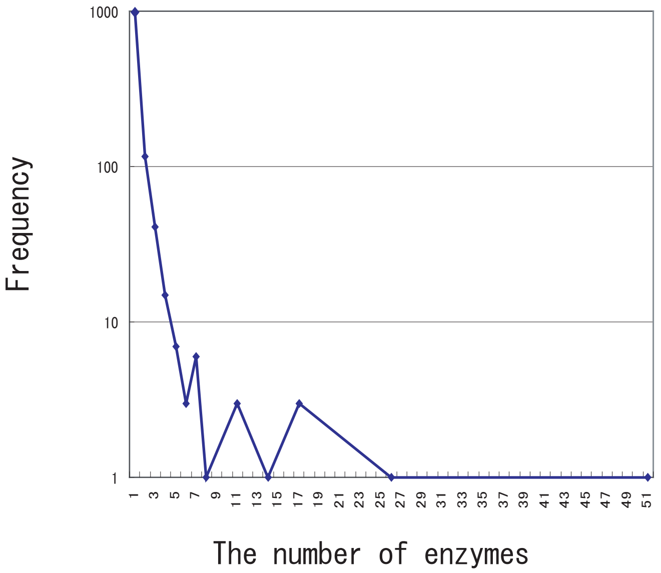

#Class	#Freq
1	981
2	116
3	41
4	15
5	7
6	3
7	6
8	1
11	3
14	1
17	3
26	1
51	1
