# Supplementary material for: Extraction of phylogenetic network modules from the metabolic network
Source: BMC Bioinformatics. 2006 Mar 13;7:130. doi: 10.1186/1471-2105-7-130 (PMC1501048; doi:10.1186/1471-2105-7-130)
Supplement: Additional File 3 — The distribution of the number of enzymes in a phylogenetic network module using Correlation coefficient with three different thresholds. [file 1471-2105-7-130-S3.pdf]

Additional file 3

The distribution of the number of enzymes in a “phylogenetic network modules” using Correlation coefficient(CC) with three different thresholds (top 1, 2.5 and 5 percentiles).

P(0010)

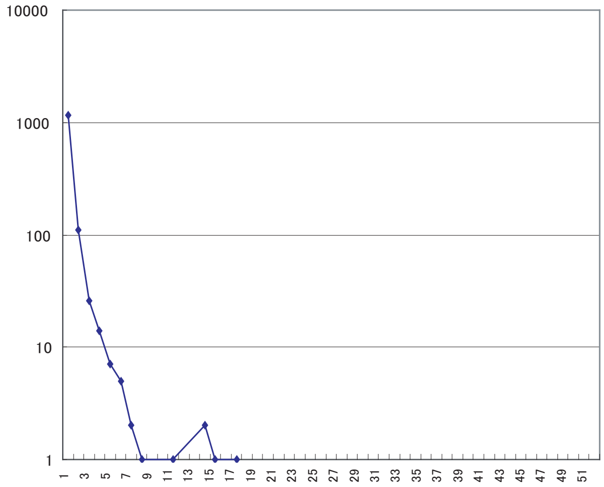

#Class	#Freq
1	1162
2	110
3	26
4	14
5	7
6	5
7	2
8	1
11	1
14	2
15	1
17	1

P(0025)

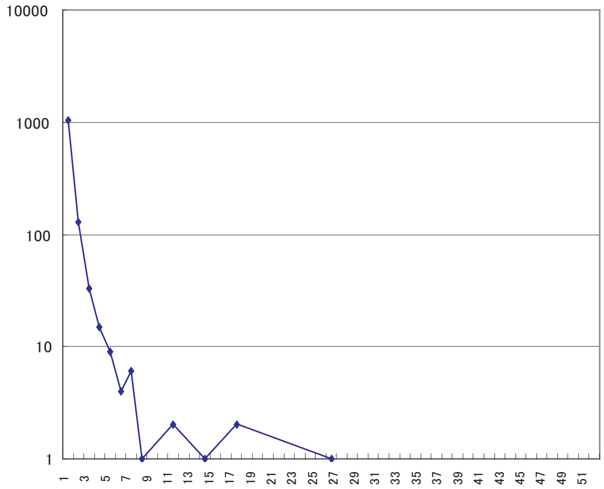

#Class	#Freq
1	1044
2	128
3	33
4	15
5	9
6	4
7	6
8	1
11	2
14	1
17	2
26	1

P(0050)

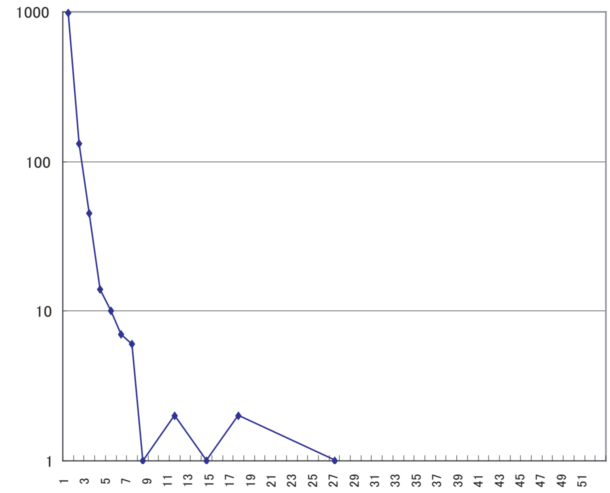

#Class	#Freq
1	983
2	131
3	45
4	14
5	10
6	7
7	6
8	1
11	2
14	1
17	2
26	1
